# Leveraging Information Technology to Improve Control of Neglected Tropical Diseases

**DOI:** 10.1371/journal.pntd.0002353

**Published:** 2013-11-07

**Authors:** Rajesh Gupta, Paul H. Wise

**Affiliations:** 1 Stanford Health Policy, Stanford University, Stanford, California, United States of America; 2 Department of Pediatrics, Stanford University School of Medicine, Stanford, California, United States of America; London School of Hygiene & Tropical Medicine, United Kingdom

## Assessing Information Technology in NTD Control

When the internet emerged to prominence in the 1990s, websites accessed from computers were mostly static and displayed information that was collected and updated at given points in time. Two decades later, mobile phones and other devices codominate the information technology (IT) space, and websites and other tools are dynamic systems that intensively incorporate instantaneous interaction among people. Individuals now use text messaging on basic mobile phones to negotiate market prices for agricultural products and social networking on advanced smartphones to coordinate mass political protests.

We argue that the impact of IT in NTD control will not be fully realized without IT tools designed to enable dynamic human interaction and real-time information exchange. In NTD control efforts, the use of IT primarily relies on computers and focuses on two areas: epidemiology and advocacy (see Table 1). Several web-based atlases and databases display the epidemiology of particular NTDs or subgroups of NTDs, often using global or country-level maps. Multiple websites raise the profile of NTDs by incorporating images, displaying key figures and information, and listing the major organizations involved in NTD control. The major deficiency in this use of IT is the primary focus on static information displays through websites, reminiscent of the 1990s' IT approach. However, recent innovations in the broader IT community (see Table 2) may reveal IT-based solutions capable of addressing two central challenges in NTD control: service delivery and research.

**Table 1:** Selected uses of information technology in NTD control efforts.

Category	Name	URL
Epidemiology	Global Atlas of Trachoma	www.trachomaatlas.org
	Global Atlas of Helminth Infections	www.thiswormyworld.org
	WHO's Global Health Repository	www.who.int/gho/neglected_diseases/en/index.html
	Global Neglected Tropical Diseases	www.gntd.org
	Atlas of Human African Trypanosomiasis	www.who.int/trypanosomiasis_african/country/foci_AFRO/en/index.html
Advocacy	Global Network for Neglected Tropical Diseases	www.globalnetwork.org
	The International Society for Neglected Tropical Diseases	www.isntd.org
	Uniting to Combat NTDs	www.unitingtocombatntds.org
	NTD NDGO Network	ntd-ngdonetwork.org
	End7	www.end7.org
	The END Fund	www.end.org

**Table 2:** Selected broader information technology approaches.

Category	Name	URL
Services Mapping	WorldBank Open Aid Partnership	wbi.worldbank.org/wbi/open-aid-partnership
	Google Maps	maps.google.com
	Ushahidi	www.ushahidi.com
Social Media Profiles	Facebook	www.facebook.com
	LinkedIn	www.linkedin.com
	ResearchGate	www.researchgate.net
Communication Platforms	Twitter	www.twitter.com
	Switchboard	www.switchboard.org
	Blogger	www.blogger.com
	Tumblr	www.tumblr.com
Cloud-based Storage	Dropbox	www.dropbox.com
	SugarSync	www.sugarsync.com
	Box	www.box.com
Cloud-based Analytics/Systems	Institute for Systems Biology	www.systemsbiology.org
	Numerate (with Merck)	www.numerate.com
	Cloudera (with the Mount Sinai School of Medicine)	www.cloudera.com
Global Labor Pool	Samasource	samasource.org
	Odesk	www.odesk.com
	Crowdflower	www.crowdflower.com
	MobileWorks	www.mobileworks.com
Financing Mechanisms	IAmScientist	www.iamscientist.com
	Microryza	www.microryza.com
	Kickstarter	www.kickstarter.com
	Fundly	www.fundly.com
Connectivity	One Laptop Per Child	one.laptop.org
	Aakash	www.aakash.org.in
	MedicMobile	www.medicmobile.org
	OpenMRS	www.openmrs.org
	O3B	www.o3bnetworks.com
	Seacom	www.seacom.mu
	Alliance for Affordable Internet	a4ai.org
	Internet.org	internet.org
	Project Loon	www.google.com/loon

## Improving Service Delivery

Because NTD control programs are often underfunded and less well integrated into other more robust health infrastructure, they have a particular need for information that permits the efficient use of resources and coordination among NTDs and related programs. This, in turn, often requires good information on the local epidemiology of the problem as well as the organizations operating in the area and the services provided. In addition, the exchange of information on best practices and protocol refinements, and the formation of real-time collaborations, can be crucial to NTD program success. An online portal, perhaps building on existing advocacy-based NTD websites while incorporating social networking components [1], could help link organizations and individuals involved at the local, district, and national levels. Using a global map interface like Google Maps, one could select a geographical location and, from road level up to country level, see all the organizations working in that location. The World Bank Open Aid Partnership, which has recently incorporated Google's Mapmaker platform into its efforts, is a notable example of this type of detailed services mapping effort. Ushahidi allows individuals to contribute various forms of media and information in real-time to map and coordinate activities. Each organization could have a profile similar to a Facebook or LinkedIn profile listing organizational information, such as services provided, data collected, dedicated staff, and sources of funding. The staff within an organization could also have individual profiles with professional information. This platform would be connected to a Twitter, Blogger, Tumblr, or Switchboard type of communication system where thoughts, ideas, strategies, and data could be rapidly shared. Instantaneous exchange of information could allow organizations to adopt and pilot different approaches much faster than current patterns of information dissemination that generally rely on meetings, conferences, and academic publications. Such an internet-based platform could have multiple benefits, including allowing individuals and organizations to collaborate more easily and rapidly, managers to task-shift and optimize their human capital, governments to improve care delivery in areas of poor infrastructure, donors to coordinate financing activities and reporting requirements, and policy makers to rapidly develop best practices [2]–[4].

## Fostering Research

NTD research efforts are relatively splintered globally and generally poorly coordinated. One mechanism to increase the number of researchers and research collaborations, particularly in resource-limited settings, is the use of a virtual model based on a singular portal linking all NTDs research. This portal could operate similarly to a Yahoo/Bing/Google search engine with the capacity to search and link "-omics" data, manuscripts, disease prevalence maps, macroeconomic data, health systems data, etc. that are currently spread across multiple nonlinkable websites and databases (some of which are also restricted via pay-for-access firewalls). Next, cloud-based analytic tools (i.e., those that perform large-scale computational activities though the internet rather than requiring a high-end computer, thus permitting such activities to be conducted on a basic personal computer or a mobile phone) could open NTD research to a much larger and more diverse community of investigators throughout the world. The Institute for Systems Biology, Numerate (in collaboration with Merck), and Cloudera (in collaboration with the Mount Sinai School of Medicine) are using cloud-based analytics to advance personalized medicine, drug development, and understanding of disease dynamics. Data integration and analytic activities could be coordinated under a single, international collaborative effort much like the Human Genome Project and the Human Microbiome Project [5]. Initial efforts, like ResearchGate, are exploring the social networking component of research that could be incorporated into this virtual model.

There are also models that benefit from the use of a global talent pool with access to basic or advanced technologies to complete a variety of tasks quickly and at relatively low cost. Samasource, ODesk, CrowdFlower, and MobileWorks focus on providing opportunities to individuals anywhere in the world to complete small to large tasks at a fast speed and low cost. This approach could facilitate outsourcing specific components of research projects, particularly to individuals or programs in resource-limited settings. Participants in resource-limited settings would benefit financially through direct payment and operationally through participation in projects. Over time, many of these entrepreneurial researchers in resource-limited settings could gain enough exposure and experience with research projects to become principal investigators and create their own research teams. Innovative financing for new researchers' involvement could potentially be raised online through science-specific initiatives such as IAmScientist or Microryza, or more broadly through Kickstarter or Fundly.

## Critical Requirements—Connectivity and Ownership

Connectivity is a product of three areas: hardware, software, and bandwidth. The gap between the group of countries with highest connectivity compared to those with lower connectivity remains clear: 63 times more access per capita to personal computers, 42 times more internet users per capita, and 25,000 times better bandwidth [6]. Mobile phones are becoming more accessible in the least developed countries but access is still lower than in developed and transition countries [7]. Furthermore, smartphone penetration rates and per capita access to computers are lower in resource-limited settings [8], [9]. Current efforts to develop highly accessible mobile and computer technologies, such as One Laptop Per Child and the Aakash 2 tablet computer (and its competitors), may offer insight into designing higher speed advanced technologies at lower cost. Most software is developed from the perspective of more advanced hardware. In contrast, MedicMobile and OpenMRS are creating patient and provider software tools to be used on the basic hardware platforms. In resource-limited settings the bandwidth is often low and sporadic. However, innovative technologies used by companies like O3B and Seacom are improving internet speed and reliability in many low-resource settings. Major IT companies and the Alliance for Affordable Internet are exploring multiple aspects of connectivity via recently launched multi-partner initiatives.

Ownership in the NTD arena has largely reflected fragmented, local hosting and monitoring. This approach can be highly inefficient and tends to undermine database integration, effective searching, and user access. An increasing trend in the IT community is the development of cloud-based systems that are monitored locally but hosted globally such that they are accessible from any location. Popular free email services rely on cloud computing, and over 20 companies like Dropbox, Sugarsync, and Box offer free storage systems in the cloud. Monitoring of NTD IT systems should remain local, but there is a strong need to move toward cloud-based systems that offer equivalent levels of security, privacy, and data restriction capacities, when needed.

Moving forward, the NTD community needs to take full advantage of innovation in the broader IT world by engaging more purposefully those who finance IT development, companies who develop and provide IT services, developers who often host "hackathon" events to create IT tools for nonprofits, and academic, IT "incubator" groups. Such actions can help ensure the design of specific IT applications to solve critical obstacles for NTD control programs. The use of IT in NTD control is just emerging, and current efforts are commendable [10]–[15]. However, the NTD community needs be at the forefront of IT innovations that could fundamentally advance NTD research and control for years to come.
